# Multi-Modal Imaging with a Toolbox of Influenza A Reporter Viruses

**DOI:** 10.3390/v7102873

**Published:** 2015-10-13

**Authors:** Vy Tran, Daniel S. Poole, Justin J. Jeffery, Timothy P. Sheahan, Donald Creech, Aleksey Yevtodiyenko, Andrew J. Peat, Kevin P. Francis, Shihyun You, Andrew Mehle

**Affiliations:** 1 Medical Microbiology and Immunology, University of Wisconsin Madison, Madison, WI 53706, USA; vgtran@wisc.edu (V.T.); dspoole@wisc.edu (D.S.P.); 2 Microbiology Doctoral Training Program, University of Wisconsin Madison, Madison, WI 53706, USA; 3 Carbone Cancer Center, University of Wisconsin Madison, Madison, WI 53706, USA; jjjeffery@wisc.edu; 4 GlaxoSmithKline, Antiviral Discovery Performance Unit, 5 Moore Drive, Research Triangle Park, NC 27709, USA; sheahan@email.unc.edu (T.P.S.); donald.r.creech@gsk.com (D.C.); andy.j.peat@gsk.com (A.J.P.); shihyun.k.you@gsk.com (S.Y.); 5 PerkinElmer, 68 Elm Street, Hopkinton, MA 01748, USA; Aleksey.Yevtodiyenko@perkinelmer.com (A.Y.); Kevin.Francis@perkinelmer.com (K.P.F.)

**Keywords:** influenza virus, reporter virus, multi-modal imaging, multiplicity reactivation, J0101

## Abstract

Reporter viruses are useful probes for studying multiple stages of the viral life cycle. Here we describe an expanded toolbox of fluorescent and bioluminescent influenza A reporter viruses. The enhanced utility of these tools enabled kinetic studies of viral attachment, infection, and co-infection. Multi-modal bioluminescence and positron emission tomography–computed tomography (PET/CT) imaging of infected animals revealed that antiviral treatment reduced viral load, dissemination, and inflammation. These new technologies and applications will dramatically accelerate *in vitro* and *in vivo* influenza virus studies.

## 1. Introduction

Influenza replication is a highly dynamic process. Yet, the majority of our knowledge about the replication cycle is extrapolated from static snapshots during an infection. This limitation can be overcome by using reporter viruses that facilitate real-time longitudinal measures of virus replication. Existing influenza reporter viruses encoding luciferases or fluorescent proteins have proven useful in rapidly measuring viral infection *in vitro* and most recently in animals [1,2,3,4,5,6,7,8,9,10,11,12,13,14]. However, some reporter viruses fail to recapitulate wild-type infection due to attenuation, genetic instability, disrupted splicing and altered gene expression [3,4,5,6,7,8,9,10,11,12,13,14]. There is thus a need for improved reporter viruses and specialized applications that probe discrete stages of viral replication and pathogenesis.

**Figure 1:**
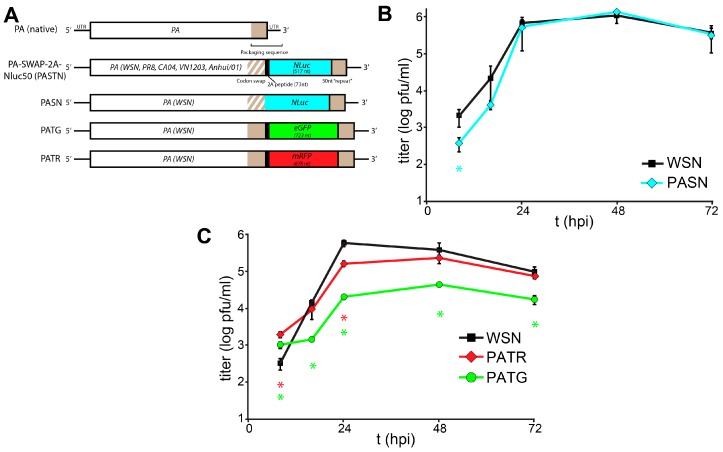
Characterization of a toolbox of influenza PA-reporter viruses. (**A**) Schematic of *PA*-reporter gene fusions encoding PA polyproteins containing the 2A peptide (PA-2A-GFP [PATG] and PA-2A-RFP [PATR]) or PA direct fusions that lack the 2A sequence (PA-SWAP-NLuc [PASN]). Silent mutations introduced into the terminal 47 nt of the PA coding sequence are depicted with diagonal lines and the 50 nt repeat of the PA open reading frame (ORF) that maintains the packaging signal is shown; (**B**,**C**) Multicycle replication kinetics of parental WSN and PA-reporter viruses in A549 cells infected at a multiplicity of infection (MOI) = 0.01. Viral titers were determined at the indicated time points by plaque assay. Data are mean of *n =* 3 ± standard deviation (sd). statistically significant differences between WSN and the reporter viruses are marked (* *p* < 0.05, Student’s *t*-test).

Recently, we developed an influenza reporter virus expressing NanoLuc (NLuc) [15] that possesses native-like replication properties and is suitable for *in vivo* imaging [1]. *NLuc* was encoded on the polymerase subunit PA gene separated by a 2A sequence to generate a “self-cleaving” polyprotein (Figure 1A). Silent mutations were introduced into the PA open reading frame (ORF) and downstream packaging signals were restored to create a PA-Swap-2A-Nluc (PASTN) virus in the strains A/WSN/33 (H1N1)(WSN) [1], the 2009 pandemic A/California/04/2009 (H1N1)(CA04) [2], A/Puerto Rico/8/1934 (H1N1), A/Vietnam/1203/2004 (H5N1), and A/Anhui/01/2013 (H7N9) (data not shown). Both PASTN and PASTN^CA04^ replicated in culture and *in vivo* with kinetics, titers, and pathogenicity remarkably similar to the parental viruses. PASTN^CA04^ was further used to track contact- and aerosol-based transmission in real time, with transmission patterns and kinetics similar to the parental strain. Thus, PASTN represents a powerful tool for quantitative, real-time, serial measures of viral replication and dissemination in culture and in animal models.

## 2. Materials and Methods

### 2.1. Plasmids, Cells and Antibodies

The influenza reverse genetics system pTM-polI-WSN-All was a kind gift from Y. Kawaoka [16]. pTMΔRNP was derived from pTM-polI-WSN-All [17]. The virus rescue constructs pBD-PB1, -PB2, -PA, and -NP express both viral RNA (vRNA) and messenger (mRNA) and have been described previously [17,18].

PA-based reporter genes were constructed following our previous approach [1]. Briefly, the *PA* open reading frame (ORF) was fused to coding sequence for *Nluc*, *mRFP* or *eGFP*. *NLuc* sequences were amplified from pNL1.1 (Promega, Madison, WI, USA). To restore the required packaging signal, the 3′ 50 nt from the *PA* ORF were repeated after the reporter genes and followed by the untranslated region from the *PA* gene. Where indicated, the 3′ 50 nt in the *PA* ORF were codon optimized (*i.e.*, “swapped”) to remove duplicate packaging signals and sequence repeats. Finally, certain reporter constructs also encoded the “self-cleaving” 2A peptide from porcine teschovirus, resulting in expression of a polyprotein that is processed following translation into distinct polypeptides for PA and the reporter. Sequences for these reporter genes have been deposited in GenBank (KT377269-KT377271). Western blots were performed with rabbit anti-PA antibodies (kindly provided by G. Zvirblis [19]).

MDCK, MDBK, 293T and A549 cells were grown in Dulbecco’s modified Eagle’s medium supplemented with 10% fetal bovine serum. All cells were grown at 37 °C in 5% CO_2_. Infections were performed in media supplemented with 25 mM 4-(2-hydroxyethyl)-1-piperazineethanesulfonic acid (HEPES), 0.3% bovine serum albumin (BSA), 1X penicillin/ streptomycin, and 0.25 to 0.5 µg/mL L-1-tosylamido-2-phenylethyl chloromethyl ketone (TPCK)-trypsin.

### 2.2. Virus Production, Infections and Attachment Assay

Virus was recovered by reverse transfection of rescue plasmids into a co-culture containing 293T and MDBK cells [1]. Viruses were subsequently amplified in MDBK cells or embryonated eggs, and titers were determined by a plaque assay performed on MDCK cells. The genotype of reporter viruses was confirmed by diagnostic reverse transcription polymerase chain reaction (RT-PCR).

Multicycle replications were initiated by infecting A549 cells at a multiplicity of infection (MOI) of 0.01 in media supplemented with 25 mM HEPES, 0.3% (BSA), 1X penicillin/streptomycin, and 0.25 to 0.5 μg/mL TPCK-trypsin. Aliquots were removed at the indicated time points and titered by a plaque assay to determine viral load. Viruses encoding fluorescent reporters were used to infect A549 cells and were visualized by fluorescence microscopy at the peak of fluorescence (18–19 hours post-infection (hpi)). Single-cycle infections to detect multiplicity reactivation were performed in MDCK cells in a 96-well format. Cells were co-infected with PASTN at an MOI of 0.005 and the indicated amount of the parental WSN. Viral gene expression was quantitated 8 hpi by performing a Nano-Glo assay (Promega) [1].

Virion attachment assays were performed with PASN that was purified by centrifugation through a 20% sucrose cushion. A549 or MDCK cells were seeded in a 96-well plate the day prior. Where noted, cells were pre-treated with 5 µL/mL of receptor-destroying enzyme supplemented with 100 µg/mL CaCl_2_ (*Vibrio cholera* filtrate, C8772, Sigma-Aldrich, St. Louis, MO, USA). Virions were added to cells, incubated for the indicated amount of time, and unbound virions were removed by washing. Bound virions were detected by performing a Nano-Glo assay (Promega) [1].

### 2.3. Mouse Infections and Imaging

Female BALB/c mice (Jackson Laboratory, Bar Harbor, ME, USA) (4 to 6 weeks old) were inoculated intranasallywith 10^3^ plaque-forming units (pfu) of PASTN in 25 µL under light isoflurane anesthesia. Body weight was monitored daily. Mice losing >20% of their original body weight were euthanized. Mice were treated with oseltamivir (BAM66450, Synchem, Elk Grove Village, IL, USA) at 10 mg/kg twice daily via oral gavage, or mock treated, beginning two days post-infection (dpi). Bioluminescent imaging and analyses were performed as described [1]. Positron emission tomography–computed tomography (PET/CT) imaging was performed on an Inveon microPET/CT using the radiotracer probe [18F]-2-deoxy-2-fluoro-d-glucose (^18^F-FDG). All animal studies were reviewed and performed in accordance with the University of Wisconsin—Madison Institutional Animal Care and Use Committee. Additionally, animal studies were conducted in accordance with the GSK Policy on the Care, Welfare and Treatment of Laboratory Animals and were reviewed the Institutional Animal Care and Use Committee at GSK.

## 3. Results and Discussion

We expanded the scope and utility of our reporter virus system by creating derivatives with the fluorescent proteins enhanced green fluorescent protein (eGFP) (PA-2A-GFP [PATG]) and monomeric red fluorescent protein (mRFP) (PA-2A-RFP [PATR]) (Figure 1A). Constructs were created following our previously published approach that maintains the complete *PA* coding sequence and duplicates sequence to recreate the contiguous packaging signals necessary for virus production [1]. Reporter constructs were also created that lacked the 2A peptide sequence, creating PA fusion proteins that mediate the specific packaging of the NLuc reporter proteins into viral particles (PA-SWAP-NLuc [PASN]). PA-based reporter viruses were rescued as described and incorporation of full-length reporter genes was verified by RT-PCR of viral stocks [1,17,20]. Silent mutations were introduced into the packaging signals contained within the *PA* ORF of most constructs on the premise that it would enhance genome stability [1]. However, these changes were not essential as both *eGFP* and *mRFP* were retained in their respective viruses without silent mutations in the packaging sequence.

Replication kinetics of the multi-label reporter virus system were characterized in human A549 lung cells (Figure 1B,C). Replication of PASN, which encodes the PA-NLuc fusion, was nearly indistinguishable from WSN. Despite the relatively large size of mRFP, PATR replicated similar to WSN, although the peak viral titer of PATR was slightly reduced compared to the parental virus. The GFP reporter virus PATG exhibited the highest degree of attenuation, and replicated similar to some other existing reporter viruses with titers >1 log below WSN. Similar to what was seen for PASTN [1], plaques for these reporter viruses were smaller and less sharply defined when compared to parental WSN. As different reporter viruses replicate with different properties, care must be taken when combining reporters in the same experiment. Perhaps unexpectedly, there was no clear correlation between native replicative capacity of the reporter virus and the size of the reporter, its fusion to PA, or if silent mutations were present in *PA*.

**Figure 2:**
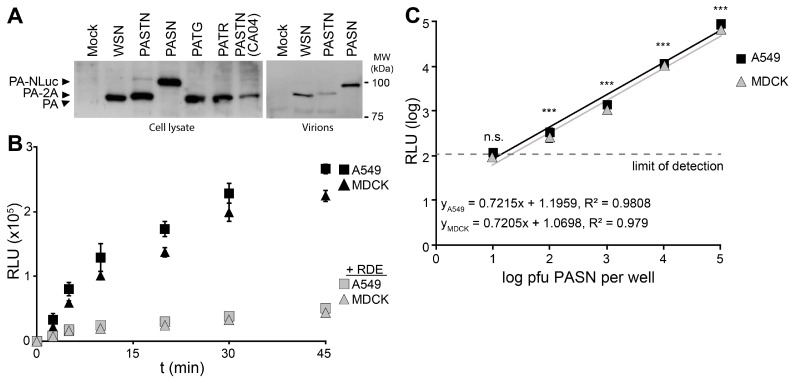
Bioluminescent virions provide a rapid and quantitative measure of attachment to cells. PASN packages PA-NLuc fusions to create bioluminescent virions. (**A**) PA fusion proteins were detected by western blotting of lysates from infected cells (left) or virions purified through a 20% sucrose cushion (right); (**B**) PASN attachment assay. 10^6^ plaque-forming units (pfu) of PASN per well were incubated with A549 or MDCK cells at 37 °C for the indicated time. Where noted, cells were pre-treated with receptor destroying enzyme (RDE) to remove sialic acids. Following washing to remove unbound virus, attached virions were measured in a 96-well NanoGlo assay. Data are mean of *n* = 3 ± sd; (**C**) A highly sensitive attachment assay. A549 or MDCK cells were incubated with dilutions of virus at 4 °C for 45 min to allow binding to go to completion. Following washing to remove unbound virus, attached virions were measured in a 96-well NanoGlo assay and the limit of detection was set to background luminescence in the absence of infection. Data are mean of *n* = 4 ± sd. Statistically significant differences between cells inoculated with PASN or media alone for each cell line are marked (* *p* < 0.001, Student’s *t*-test).

Reporter viruses were used to study early stages in the viral life cycle, beginning with attachment using bioluminescent PASN virions. Hemagglutinin (HA)-mediated attachment to sialic acids on the cell surface is essential during infection and is often disrupted by HA-specific antibodies [21]. Western blotting of PA from infected cells showed that all of the reporter viruses expressed PA at similar level, regardless of the nature of the reporter protein or whether it remained fused to PA (Figure 2A). Blotting of purified virions showed that direct fusion of NLuc to PA resulted in specific packaging of PA-NLuc into PASN virions creating bioluminescent particles, but not the polyprotein of PASTN virions (Figure 2A). To measure the kinetics of virion binding in a high-throughput setting, PASN was incubated with A549 or MDCK cells in a 96-well format (Figure 2B). In parallel, assays were performed on cells pre-treated with neuraminidase to remove sialic acids. At the indicated times, free virions were removed by washing and bound virions were detected by performing a NanoGlo luciferase assay. As the NLuc reporter is packaged within the virion, these assay are designed to measure virion binding to cells, and not infection *per se*. Binding to A549 and MDCK cells proceeded rapidly and reached a maxima in ~45 min, paralleling prior analyses [22]. Furthermore, neuraminidase treatment reduced binding to background levels indicating that virion binding, and resultant luciferase activity, required sialic acids on the cell surface. To determine the sensitivity of the binding assay, we repeated the experiments with decreasing amounts of virions and allowed binding to proceed to completion (Figure 2C). Our data show a strong linear relationship over 4 logs between input and bound virions. Binding by as few as 100 pfu yielded a reproducible and statistically significant signal above background. This is a highly sensitive readout considering that this assay directly measures virion binding, and does not benefit from the signal amplification that occurs when infections are allowed to proceed to viral gene expression or replication. The entire binding assay was completed in under an hour, demonstrating that bioluminescent PASN particles can be used for the rapid and specific measurement of virion attachment without the need for virus replication. This assay could be easily adapted to measure receptor binding specificity or the ability of neutralizing antibodies or similar therapeutics to block attachment.

**Figure 3:**
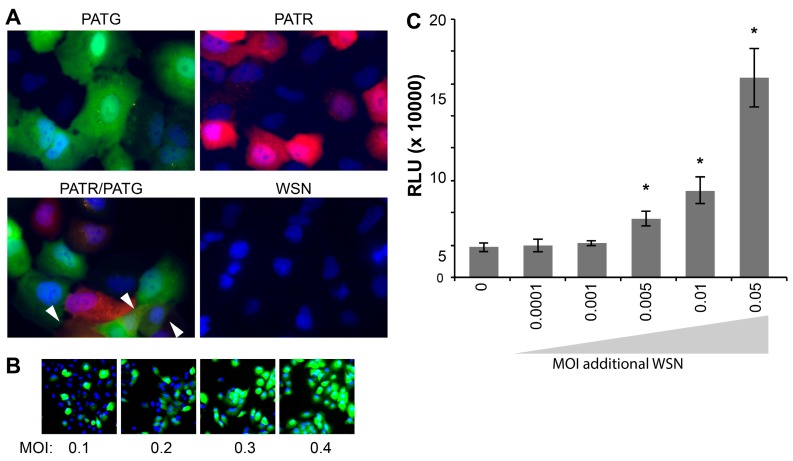
Influenza reporter viruses detect infection, co-infection, “semi-infection” and multiplicity reactivation. Live cell imaging of viral gene expression in infected cells. (**A**) A549 cells were infected with PATG, PATR, WSN or co-infected with PATG and PATR. Infected cells were detected by fluorescence microscopy 18–19 hours post-infection (hpi) and cells were counter-stained with Hoechst 33342 to detect nuclei. Co-infection is indicated by the arrowheads; (**B**) A549 cells were infected with an increasing MOI of PATG. Cells were imaged to detect total cells (Hoechst 33342) and those expressing a virally encoded gene (*GFP*). All of the cells are GFP-positive when only an MOI = 0.4 is used; (**C**) Multiplicity reactivation rescues viral gene expression. A total of 25,000 MDCK cells were infected in a 96-well format with PASTN at an MOI = 0.005. Where indicated, cells were also simultaneously infected with increasing amounts of WSN. A luciferase activity assay was performed 10 hpi to determine *PASTN* gene expression for each condition (average relative light units (RLU), Data are mean of *n* = 4 ± sd). Conditions with a statistically significant increase in gene expression compared to PASTN alone are marked (* *p* < 0.05, Student’s *t*-test).

Following attachment and entry, fluorescent reporter viruses are especially useful in tracking gene expression at the cellular level without the administration of exogenous substrate for reporter activity. Live-cell microscopy detected robust fluorescence in A549 cells infected with PATG and PATR, but not WSN (Figure 3A). Singly infected and co-infected cells were simultaneously detected in cultures exposed to both PATG and PATR.

Whereas many assays, such as plaque assays and the determination of the 50% Tissue Culture Infective Dose (TCID_50_), rely on successful infection and completion of the viral life cycle, our fluorescent and bioluminescent viruses report on gene expression, and not necessarily the capacity to produce infectious particles. It has recently been suggested that a large percentage of influenza virions are capable of initiating infections, but cannot support production of infectious progeny, so-called “semi-infectious” virions [23]. As such, these semi-infectious virions are not detected in viral titer measurements that rely on multiple rounds of infection, including the plaque assays used here to titer our viral stocks. We exploited the fact that PATG reports on viral gene expression to test for the presence of semi-infectious particles during early stages of infection (Figure 3B). Cells were infected with increasing amounts of virus and imaged to detect total cells (Hoechst 33342) and those expressing a virally encoded gene (*GFP*). These experiments routinely detected 3–5 fold more cells expressing virally encoded *GFP* than would be predicted based on the MOI used to initiate the infection; all of the cells inoculated at an MOI = 0.4 were GFP-positive, when only ~33% are predicted to be infected assuming an ideal Poisson distribution (Figure 3B). This disconnect between MOIs based on pfu and the number of GFP-positive cells in the resultant infection suggests that our reporters detect a large number of semi-infectious events that initiate infections and gene expression, but cannot produce infectious progeny.

Multiplicity reactivation predicts that semi-infectious events can be complemented during co-infection to restore viral gene expression and potentially virus production [24]. To address this possibility quantitatively and provide further support for the largely qualitative results obtained with PATG, cells were co-infected with PASTN and non-reporter WSN in an attempt to restore NLuc expression from semi-infectious reporter viruses. Single-cycle infections were initiated with a very low MOI of PASTN (0.005) in the presence of increasing amounts of the non-reporter WSN (Figure 3C). Addition of WSN increased expression of PASTN-encoded NLuc upwards of 3-fold, suggesting either co-infection by fully infectious particles or complementation of defective PASTN by the parental WSN. Strikingly, complementation still occurred when both viruses were present at an MOI of 0.005, conditions where co-infection by fully infectious particles is highly unlikely (*i.e*., using viral titers determined by plaque assays to establish the inoculum, less than 1 cell out of the 25,000 total per well is predicted to be co-infected by fully infectious particles when both viruses are at an MOI of 0.005). This suggests that some of the enhanced gene expression for PASTN resulted from complementation during co-infection by two “semi-infectious” particles. Moreover, as WSN enhanced NLuc expression which was only present in the reporter virus genome, these data provide evidence that one mode of multiplicity reactivation occurs by restoring expression from an otherwise inactive gene segment, as opposed to solely supplying a replacement for the defective segment. By exploiting the unique advantages of these reporter viruses, we have established methods to quantitate semi-infectious events and multiplicity reactivation, and probe their potential role during infection and viral evolution.

**Figure 4:**
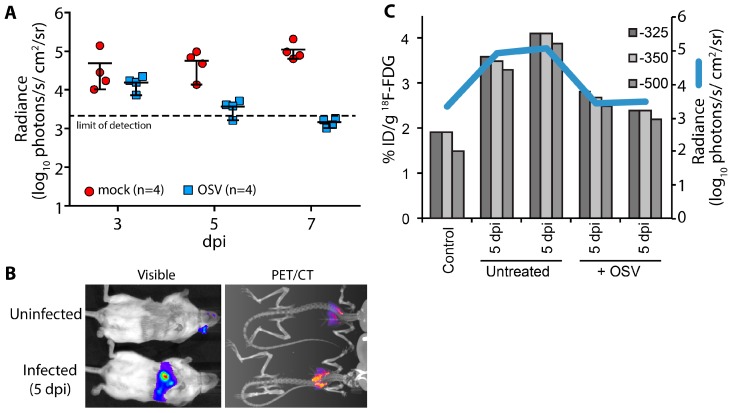
Multi-modal imaging of the effect of antiviral treatment on influenza virus-mediated inflammation. (**A**) Infected mice were treated with oseltamivir (OSV, 10 mg/kg bid) beginning 2 days post infection (dpi) or mock treated; Serial bioluminescence imaging of the same cohort of animals was used to detect statistically significant differences in viral load over time between mock and OSV treatment (*p* = 0.0085, 2-way ANOVA); (**B**) Multi-modal bioluminescence and positron emission tomography–computed tomography (PET/CT) imaging. [18F]-2-deoxy-2-fluoro-d-glucose (^18^F-FDG) was used as a probe to measure inflammation in the lungs. Representative data from uninfected and infected mice are shown; (**C**) Dual quantification of viral load (bioluminescent radiance) and inflammation (%-injected dose(ID)/g) in the lungs of infected or control mice. Three different Hounsfield unit thresholds were used to define lung tissue by CT from which ^18^F-FDG was quantified by PET. Data were collected from one control mouse, two infected mice, and two infected mice treated with OSV.

A key advantage of using non-invasive reporters is the capacity for longitudinal measures throughout the course of infection and sequential measures using different imaging modalities or spectra. Using longitudinal bioluminescent imaging to measure virus replication and tissue distribution in the same animals, we evaluated the antiviral kinetics of the neuraminidase inhibitor oseltamivir (OSV). Animals were treated beginning 2 days post PASTN inoculated and viral load was quantified by *in vivo* imaging (Figure 4A). In contrast to untreated animals, virus replication was significantly reduced with OSV treatment (*p* = 0.0085, 2-way ANOVA) and was below the limit of detection by 7 days post infection (dpi), preventing an otherwise lethal infection.

We then paired bioluminescence and PET/CT imaging to measure both virus replication and inflammation. Inflammation was measured in a subset of mice at 5 dpi using ^18^F-FDG, a radiotracer for detecting influenza-mediated inflammation [25] (Figure 4B). Lungs were segmented from computed tomography (CT) data using three Hounsfield unit thresholds to prevent artifacts due to changes in lung tissue density from edema and immune infiltrates. Inflammation was then measured from the positron emission tomography (PET) data by quantifying ^18^F-FDG localized to each of the CT-defined lung volumes (Figure 4C). OSV treatment dramatically reduced inflammation compared to mock-treated animals, approaching background levels detected in an uninfected control. Multi-modal imaging enabled measurement of both viral replication and host responses, and showed a remarkable correlation between viral load and inflammation in mice.

In conclusion, we report the generation of a versatile toolbox of influenza A reporter viruses. These viruses are suited for probing discrete stages of the viral replication cycle at the cellular level—from attachment to the cell, through gene expression, and ultimately the spread of released virus. Moreover, multi-modal *in vivo* imaging experiments demonstrate the ability to simultaneously measure viral replication, the efficacy of antiviral or immunomodulatory therapy, the host antiviral response, and the close interdependence of these variables.
